# Thermographic analysis of the periorbital region in mouth and nasal breathing children

**DOI:** 10.1590/2317-1782/e20240151en

**Published:** 2025-02-21

**Authors:** Júlia Ana Soares Silva, Amanda Freitas Valentim, Yasmim Carvalho Telson, Patrícia Vieira Salles, Mariana Souza Amaral, Ana Cristina Côrtes Gama, Letícia Paiva Franco, Andréa Rodrigues Motta, Renata Maria Moreira Moraes Furlan

**Affiliations:** 1 Faculdade de Medicina, Universidade Federal de Minas Gerais – UFMG - Belo Horizonte (MG), Brasil.; 2 Departamento de Fonoaudiologia, Faculdade de Medicina, Universidade Federal de Minas Gerais – UFMG - Belo Horizonte (MG), Brasil.; 3 Programa de Pós-graduação em Ciências Fonoaudiológicas, Universidade Federal de Minas Gerais – UFMG - Belo Horizonte (MG), Brasil.; 4 Departamento de Fonoaudiologia, Pontifícia Universidade Católica de Minas Gerais – PUC Minas - Belo Horizonte (MG), Brasil.; 5 Departamento de Otorrinolaringologia, Faculdade de Medicina, Universidade Federal de Minas Gerais – UFMG - Belo Horizonte (MG), Brasil.

**Keywords:** Thermography, Eyelids, Mouth Breathing, Speech, Language and Hearing Sciences, Eye

## Abstract

**Purpose:**

To verify whether the breathing mode interferes with surface periorbital temperatures and propose a thermographic analysis method for this region.

**Methods:**

Exploratory, observational, cross-sectional study. Thermographic images of 14 mouth-breathing and 14 nasal-breathing children were collected and analyzed using the Visionfy program (Thermofy®, Brazil) with breast 1 color scale. The ellipse tool delimited the region of interest (periorbital region) and a customized shape subdivided the region of interest into four arcs. The study collected maximum, mean, and minimum absolute and standardized temperatures of the arcs and performed interrater and intrarater comparisons and comparative analysis of temperatures between groups. Temperatures in the group of mouth breathers were compared with pruritus, hyperemia, tearing, and ocular edema.

**Results:**

the intrarater agreement indicated a satisfactory result for most analyzed temperatures. The interrater agreement, in general, was excellent for maximum, good for mean, and poor for minimum temperatures. The association between periorbital data and temperatures showed an association between ocular edema and temperatures in the upper and lower right arcs and between pruritus and the maximum temperature in the lower right arc.

**Conclusion:**

the method had satisfactory intrarater and interrater agreement for maximum and mean temperatures. Ocular edema was associated with temperatures in the group of mouth breathers. Breathing mode was not associated with periorbital temperature.

## INTRODUCTION

Breathing is a function of the stomatognathic system that greatly influences the person’s overall development; hence, changes in this function can affect craniofacial, cognitive, and behavioral development^(1)^. Mouth breathing (MB) occurs when the nasal breathing (NB) pattern is replaced by oral or mixed breathing for 6 or more months^(2,3)^. Various factors can lead to this breathing pattern, including nasal obstructions, allergic disorders, physiological/anatomical changes, and genetic factors^(2,3)^. The primary etiology is allergic rhinitis, followed by adenoid hypertrophy, tonsillar hypertrophy, and obstructive nasal septum deviation^(2)^.

The main signs and symptoms include reduced orofacial muscle tone, fatigue, irritability, halitosis, daytime sleepiness, and changes in speech, chewing, and swallowing functions^(4)^. Thus, children with MB require a multidisciplinary evaluation for accurate diagnosis and treatment^(4)^. In addition to these commonly found signs and symptoms, sleep quality changes in children with MB also require attention due to the prevalence of conditions such as snoring, open-mouth sleeping, sleep apnea, drooling, and fragmented sleep^(5)^.

Speech-language-hearing assessment of children with MB requires specific tests, which may be qualitative or quantitative – although qualitative ones predominate, such as the evaluator’s observation of myofunctional changes^(1)^. New technologies, including infrared thermography, have been suggested as potentially interesting tools to assist speech-language-hearing pathologists in evaluation. This non-invasive resource allows professionals to monitor the microcirculatory activity of the skin surface quickly, safely, and painlessly, identifying inflammatory processes and changes in the endocrine, vascular, nervous, and musculoskeletal systems^(1,6,7)^.

Periorbital skin is the thinnest on the human body and is vascularized by a complex network that includes the internal carotid artery (which branches into the ophthalmic artery) and the external carotid artery (which branches into the infraorbital, facial, and superficial temporal arteries). The venous drainage of the eyelid is performed by the superficial temporal, angular, and facial veins^(8)^. Studies have shown that poor sleep generates oxidative stress and correlates with skin changes and aging. This is commonly associated with periorbital hyperpigmentation, defined as venous stasis discoloring the periorbital region^(9,10)^. This sign is also frequently observed in children with MB^(10)^.

Thus, this study aimed to verify whether the respiratory mode interferes with the surface periorbital temperature and propose a thermographic analysis method for this region. The study hypothesis is that there is a difference in periorbital temperature between MB and NB individuals, with the latter having higher temperatures in this region due to better blood supply^(9)^.

## METHODS

This exploratory, observational, cross-sectional study was approved by the Research Ethics Committee of the Federal University of Minas Gerais (UFMG) under protocol number 3.695.491. All parents or legal guardians of the children participating in the study signed an informed consent form.

The study included 28 children aged 4 to 11 years (mean age: 7.62 years, standard deviation: 2.19 years) of both sexes, divided into two groups:

1- MB Group: 14 MB children (10 males, four females).
2- NB Group: 14 NB children (10 males, four females).

MB children were recruited from the Mouth-Breather Outpatient Clinic at the UFMG Hospital. NB children were recruited from the Speech-Language-Hearing Clinic at the same hospital, among those being treated in language and audiology, as well as through the researchers’ direct contact.

Inclusion criteria for both groups were being 2 to 11 years old and responding to medical survey protocols and/or clinical exams to identify MB children^(2)^.

Exclusion criteria for both groups were wearing facial adornments or bandages; having taken a bath or used a hairdryer 2 hours before the exam; having applied facial products on the day of the exam; consuming caffeine 4 hours or eating 2 hours before the exam; having physical, neurological, or cognitive changes that hindered collaboration during the exam; using a mobile phone near the face 2 hours before the exam; engaging in physical exercises, acupuncture, massages, electrical stimulation, or prolonged sun exposure on the exam day^(11)^; and having injuries, inflammation, or scarring in the region of interest (ROI). Additionally, children who had used nasal vasoconstrictors or corticosteroids on the day of data collection were excluded. These criteria were assessed through a questionnaire administered to parents.

Children were classified as MB based on the multidisciplinary team diagnosis at the Mouth Breather Outpatient Clinic of the Clinics Hospital at UFMG. This team consisted of otorhinolaryngologists, an allergist, an orthodontist, and a speech-language-hearing pathologist. Patients were classified as obstructive when they had adenoid hypertrophy (i.e., an adenoid occupying more than 70% of the nasopharyngeal airway space)^(12)^, with or without tonsillar hypertrophy. This classification also included children with hypertrophied inferior turbinates occupying more than 50% of the nasal cavity^(2)^, as visualized through fiberoptic nasopharyngoscopy and rhinoscopy. Patients were classified as allergic when they had a positive skin test (puncture test or prick test)^(13)^. Data on MB cause (airway obstruction and/or allergies) were collected from the MB children’s medical records, along with information on the periorbital region, including the presence of hyperemia, tearing, edema, and ocular pruritus. These periorbital findings are documented during otorhinolaryngological evaluations.

Children were classified as NB with the NB feasibility test^(14)^. This involved observing the positioning of the lips during the child's habitual posture, ensuring that the child could maintain lip seal without tension. They were also identified through the medical history survey and MB clinical examination protocol^(2)^. According to this protocol, a child is identified as NB if they have, at most, one major sign or one major sign combined with one minor sign. Moreover, the Sleep Disturbance Scale for Children^(15)^ was employed to ensure that NB children had no associated sleep disorders, with a required score of 39 or less.

Data were collected at the Observatory for Speech-Language-Hearing Functional Health at the UFMG Medical School, in a controlled environment at 20 °C to 23 °C and 40% to 70% relative humidity, as recorded by a Testo^®^ thermal hygrometer, model 622. Patients remained in the environment for 15 to 20 minutes before the thermographic evaluation to allow for temperature stabilization. Their tympanic temperature was measured using a Prosnubl^®^ thermometer to standardize temperature values. The study's standardization conditions were based on the practical guide of the American Academy of Thermology^(11)^.

The thermographic evaluation used the FLIR A315^®^ camera, whose specifications are described in Chart 1^(16)^.

**Chart 1:** Specifications of the FLIR A315 camera

Model	Flir A315
Lens Focal Length	18 mm
Resolution	320 x 240 pixels
FOV	25° x 18.8°
IFOV	1.36 mrad
Spectrum Range	7.5 to 13 μm
Temperature Measurement Range	-20 °C to 120 °C / 0 to 350 °C
Uncertainty	±2 °C or ± 2%

Caption: FOV = Field of view; IFOV = instantaneous field of view

The children sat on a chair for the thermographic assessment, with their legs aligned to the floor, at a 90° angle to the trunk, and their heads in a habitual position. They were asked to wear a hair tie and/or cap, remove any adornments such as earrings and necklaces, and, obligatorily, remove the facial protective mask.

The camera was placed on a tripod, with a 90° angle to the floor, 1 meter from the chair backrest, and adjustable in height according to the child's stature. Thermal images were repeated three times with the child in a frontal position with the lips in a habitual position.

The collected measures were stored in a private folder on the computer’s memory, and the data were transferred to an Excel spreadsheet. The images were analyzed using the Visionfy software (Thermofy^®^, Brazil), considering the emissivity of human skin as 0.98^(11)^ and selecting the “breast 1” color scale to clearly visualize the area of interest, with a temperature window ranging from 25 °C to 37 °C. In this color palette, light gray represents cooler temperatures, dark gray and black represent medium temperatures, and purple and yellow represent higher temperatures.

**Figure 1:**
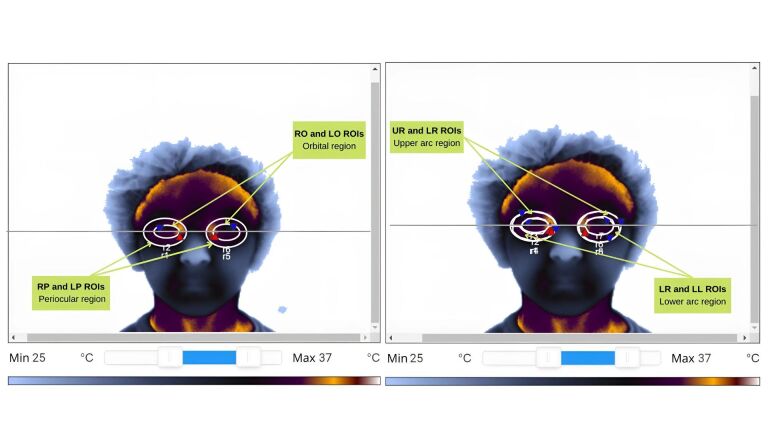
Definition of the areas of interest

The ROI (periorbital region) was selected in the thermograms using the ellipse tool (which obtains the mean temperature within an oval/elliptical region, whose perimeter passes through specific reference points) and the custom shape tool (which delineates the periorbital region). The following limits were adhered to for selecting the periorbital region (Figure 1): upper limit (region of the eyebrow), lower limit (insertion of the lower eyelid below the lower orbital rim, where the eyelid meets the denser cheek tissue, visible as a darker area), and lateral limit (darker area outside the lateral and medial canthi – the connection between the upper and lower eyelids)^(8,17)^.

Caption: ROI = region of interest; RP = right periorbital region; LP = left periorbital region; RO = right orbital region; LO = left orbital region; UR = upper right orbital arc; UL = upper left orbital arc; LR = lower right orbital arc; LL = lower left orbital arc

After defining the periorbital area, a horizontal line was drawn across the lateral and medial canthi, inserting an ellipse to mark ROI 1. This was represented by the right (RP ROI) and left (LP ROI) periorbital regions, serving as a reference to insert other shapes, along with the horizontal line. ROI 2 was also marked, represented by the right (RO ROI) and left (LO ROI) orbital regions, excluding eye temperatures.

Based on this selection, the other ROIs were marked using the custom shape tool: the upper arc of the right and left orbits (UR ROI and UL ROI, respectively) and the lower arc of the right and left orbits (LR ROI and LL ROI, respectively).

After marking the shapes, the maximum, mean, and minimum absolute temperatures and standardized temperatures for the RU, LU, RL, and LL ROIs were obtained separately. Standardized temperatures were calculated to standardize the values based on the relationship between human metabolism and the environmental conditions to which the body was exposed, using the standardized temperature equation^(18)^ (Chart 2). Its values ranged from 0 to 1, enabling homogeneous analysis and avoiding interference from environmental temperature in the results.

**Chart 2:** Standardized temperature equation

Standardized temperature =	Mean temperature at the point of interest – Environmental temperature
	Tympanic temperature – Environmental temperature

Two independent evaluators marked the area of interest and measured the temperatures, both replicating 20% of the analyses at two different times.

The descriptive analysis used the mean and standard deviation of ROI temperatures. Data were analyzed using Stata software version 13. A comparative analysis of age and temperatures between groups was performed using the Mann-Whitney test (non-parametric test). The Kruskal-Wallis test compared temperatures with symptoms such as pruritus, hyperemia, tearing, and ocular edema in the MB group. The significance level for all analyses was set at 5%. The interrater and intrarater comparisons used the intraclass correlation coefficient (ICC), classified as poor (< 0.5), moderate (0.5 to < 0.75), good (0.75 to < 0.9), and excellent (≥ 0.9)^(19)^.

## RESULTS

The sample had no statistically significant age difference between the MB and NB groups, as indicated in Table 1.

**Table 1:** Age comparison between mouth and nasal breathers

**Group**	**Mean**	**Standard deviation**	**Median**	**p-value**
NB	7.81	2.26	8.20	0.549
MB	7.43	2.19	7.95	
Total	7.62	2.19	8.00	0.549

Caption: NB = nasal breather; MB = mouth breather; p = probability of significance (Mann-Whitney test)

The MB group had seven allergic children, two with obstructive issues, four with mixed conditions (obstructive and allergic), and one classified as habitual. Additionally, periorbital data indicated that 11 children had ocular pruritus, seven had hyperemia, six had tearing, and four had edemas.

The interrater agreement is described in Table 2. The maximum temperatures had moderate to excellent agreement, the mean temperatures had poor to good agreement, and the minimum temperatures had poor to moderate agreement.

**Table 2:** Interrater comparison of temperature measures (ºC)

**Temperature measures (ºC)**	**ICC**	**Degree of correlation**
Maximum UR ROI	0.984	Excellent
Minimum UR ROI	0.084	Poor
Mean UR ROI	0.847	Good
Maximum LR ROI	0.730	Moderate
Minimum LR ROI	0.208	Poor
Mean LR ROI	0.474	Poor
Maximum UL ROI	0.998	Excellent
Minimum UL ROI	0.475	Poor
Mean UL ROI	0.859	Good
Maximum LL ROI	0.687	Moderate
Minimum LL ROI	0.502	Moderate
Mean LL ROI	0.616	Moderate

Caption: ICC = intraclass correlation coefficient; ROI = region of interest; UR = upper right orbital arc; UL = upper left orbital arc; LR = lower right orbital arc; LL = lower left orbital arc

In the intrarater agreement (Table 3), evaluator 1 had good to excellent agreement for maximum temperatures, while mean and minimum temperatures ranged from poor to excellent agreement. Evaluator 2 had moderate to excellent agreement for maximum and mean temperatures, while minimum temperatures had good to moderate agreement.

**Table 3:** Intrarater comparison of temperature measures

**Temperature measures**	**Evaluator 1 (n = 6)**		**Evaluator 2 (n = 6)**	
	**ICC**	**Degree of correlation**	**ICC**	**Degree of correlation**
Maximum UR ROI	0.998	Excellent	0.998	Excellent
Minimum UR ROI	0.977	Excellent	0.706	Moderate
Mean UR ROI	0.976	Excellent	0.958	Excellent
Maximum LR ROI	0.905	Excellent	0.744	Moderate
Minimum LR ROI	0.870	Good	0.814	Good
Mean LR ROI	0.935	Excellent	0.629	Moderate
Maximum UL ROI	0.998	Excellent	0.992	Excellent
Minimum UL ROI	0.101	Poor	0.513	Moderate
Mean UL ROI	0.351	Poor	0.959	Excellent
Maximum LL ROI	0.896	Good	0.714	Moderate
Minimum LL ROI	0.966	Excellent	0.544	Moderate
Mean LL ROI	0.953	Excellent	0.514	Moderate

Caption: ICC = intraclass correlation coefficient; ROI = region of interest; UR = upper right orbital arc; UL = upper left orbital arc; LR = lower right orbital arc; LL = lower left orbital arc

Temperatures were also compared between the MB and NB groups (Table 4). A significant difference was found only for the standardized minimum UL ROI temperature. The largest temperature difference between groups occurred for the minimum UL ROI (with a difference of 0.69°C between the MB and NB groups) and LL ROI temperatures (with a difference of 0.62°C).

**Table 4:** Distribution of temperature measures (°C) of the groups of oral and nasal breathers

**Temperature measures**	**MB Group**		**NB Group**		**p-value**
	**Mean**	**Standard deviation**	**Mean**	**Standard deviation**	
Maximum UR ROI	35.22	0.63	35.50	0.55	0.265
Minimum UR ROI	32.50	0.49	32.71	0.68	0.461
Mean UR ROI	33.81	0.51	34.12	0.55	0.167
Maximum standardized UR ROI	0.93	0.04	0.94	0.03	0.370
Minimum standardized UR ROI	0.75	0.03	0.76	0.04	0.395
Mean standardized UR ROI	0.84	0.03	0.85	0.02	0.160
Maximum LR ROI	34.64	0.85	34.94	0.70	0.270
Minimum LR ROI	31.95	1.03	32.21	0.84	0.490
Mean LR ROI	33.43	0.85	33.64	0.55	0.635
Maximum standardized LR ROI	0.89	0.05	0.90	0.04	0.358
Minimum standardized LR ROI	0.71	0.06	0.72	0.04	0.709
Mean standardized LR ROI	0.81	0.04	0.82	0.02	0.865
Maximum UL ROI	35.37	0.47	35.47	0.42	0.804
Minimum UL ROI	32.04	0.79	32.73	0.70	0.062
Mean UL ROI	33.73	0.58	34.12	0.50	0.085
Maximum standardized UL ROI	0.94	0.03	0.94	0.03	0.991
Minimum standardized UL ROI	0.72	0.04	0.76	0.04	0.038*
Mean standardized UL ROI	0.83	0.03	0.85	0.02	0.101
Maximum LL ROI	34.99	0.78	35.03	0.59	0.972
Minimum LL ROI	31.49	0.93	32.11	0.87	0.125
Mean LL ROI	33.44	0.91	33.62	0.53	0.769
Maximum standardized LL ROI	0.91	0.04	0.91	0.04	0.692
Minimum standardized LL ROI	0.68	0.05	0.72	0.05	0.087
Mean standardized LL ROI	0.81	0.05	0.82	0.03	0.865

* p ≤ 0.05

Caption: NB = nasal breather; MB = mouth breather; ROI = region of interest; UR = upper right orbital arc; UL = upper left orbital arc; LR = lower right orbital arc; LL = lower left orbital arc; p = probability of significance (Mann-Whitney test)

Table 5 shows the association between absolute and standardized temperatures and pruritus, hyperemia, tearing, and ocular edema in the MB group. Ocular edema was associated with the minimum and mean absolute UR ROI temperatures and the maximum and mean absolute and maximum standardized LR ROI temperatures. Moreover, ocular pruritus was associated with the maximum standardized UR ROI temperature.

**Table 5:** Association between temperature measures and ocular edema, ocular pruritus, ocular hyperemia, and tearing

Temperature measures	Ocular edema			Ocular pruritus			Ocular hyperemia			Tearing		
	Mean	SD	p-value	Mean	SD	p-value	Mean	SD	p-value	Mean	SD	p-value
Maximum UR ROI	35.67	0.18	0.066	35.35	0.59	0.139	35.37	0.68	0.277	35.29	0.71	0.796
Minimum UR ROI	32.89	0.16	0.048*	32.50	0.52	0.938	32.55	0.51	0.655	32.51	0.55	0.897
Mean UR ROI	34.22	0.19	0.048*	33.89	0.51	0.312	33.89	0.54	0.565	33.83	0.57	0.796
Maximum standardized UR ROI	0.95	0.01	0.203	0.94	0.03	0.043*	0.93	0.03	0.225	0.94	0.05	0.519
Minimum standardized UR ROI	0.76	0.01	0.090	0.75	0.03	0.938	0.75	0.02	0.949	0.75	0.03	0.796
Mean standardized UR ROI	0.85	0.01	0.066	0.84	0.03	0.052	0.84	0.03	0.406	0.84	0.04	0.796
Maximum LR ROI	35.33	0.45	0.034*	34.75	0.88	0.243	34.87	1.04	0.142	34.61	0.96	0.897
Minimum LR ROI	32.45	0.84	0.258	31.97	1.08	0.586	32.13	1.03	0.338	31.95	1.03	0.897
Mean LR ROI	34.11	0.32	0.034*	33.52	0.88	0.312	33.67	0.99	0.142	33.45	0.97	0.897
Maximum standardized LR ROI	0.92	0.03	0.034*	0.90	0.05	0.073	0.90	0.06	0.142	0.89	0.06	0.699
Minimum standardized LR ROI	0.73	0.06	0.396	0.71	0.06	0.697	0.72	0.06	0.565	0.71	0.06	0.897
Mean standardized LR ROI	0.84	0.02	0.066	0.82	0.04	0.139	0.82	0.05	0.180	0.81	0.05	0.699
Maximum UL ROI	35.66	0.23	0.120	35.42	0.46	0.243	35.54	0.42	0.142	35.47	0.42	0.699
Minimum UL ROI	32.58	0.46	0.090	31.99	0.72	0.484	32.31	0.61	0.225	32.00	0.59	0.606
Mean UL ROI	34.11	0.24	0.120	33.73	0.51	0.586	33.84	0.55	0.482	33.69	0.50	0.366
Maximum standardized UL ROI	0.94	0.02	0.322	0.94	0.03	0.073	0.94	0.02	0.180	0.95	0.03	0.245
Minimum standardized UL ROI	0.74	0.02	0.120	0.72	0.04	0.586	0.73	0.03	0.180	0.72	0.04	0.796
Mean standardized UL ROI	0.84	0.01	0.203	0.83	0.02	0.697	0.83	0.03	0.565	0.83	0.03	1.000
Maximum LL ROI	35.53	0.41	0.089	35.00	0.85	0.815	35.17	0.99	0.179	34.88	0.94	0.518
Minimum LL ROI	31.81	0.33	0.671	31.38	0.90	0.312	31.52	0.88	0.949	31.34	1.05	0.699
Mean LL ROI	33.93	0.47	0.203	33.45	0.95	0.815	33.55	1.13	0.406	33.35	1.13	0.519
Maximum standardized LL ROI	0.94	0.03	0.157	0.92	0.05	0.312	0.92	0.05	0.180	0.91	0.05	0.796
Minimum standardized LL ROI	0.69	0.02	1.000	0.68	0.06	0.312	0.68	0.04	0.565	0.67	0.07	0.519
Mean standardized LL ROI	0.83	0.03	0.396	0.81	0.05	0.484	0.81	0.06	0.406	0.81	0.06	0.796

* p ≤ 0.05

Caption: p = probability of significance (Kruskal-Wallis test) ROI = region of interest; UR = upper right orbital arc; UL = upper left orbital arc; LR = lower right orbital arc; LL = lower left orbital arc; SD = standard deviation

## DISCUSSION

This study developed a method for recording and analyzing periorbital thermographic images and obtaining surface temperatures of MB and NB children. Ophthalmology researchers have increasingly used orbital and periorbital thermography to diagnose inflammatory processes, eye diseases, and allergic conditions and identify eye temperature patterns^(20)^. The advantage of this method lies in using an indirect temperature measurement tool, which, due to the lack of contact with the patient, does not cause discomfort or interfere with temperature readings^(7)^.

The method used in this study to determine ROIs allowed the researchers to assess only the periorbital region, carefully excluding adjacent structures that could influence the temperatures, which are cooler in the orbit and eyelashes^(21)^. Studies have shown that the central region of the orbital surface is the coolest because it has low vascularization and its temperature is influenced by the eye’s natural lubrication and the surrounding environment^(20)^.

The ROIs analyzed in this study were based on the participants’ facial anatomy. The use of the “breast 1” color scale provided better ROI visualization, closely resembling the anatomical image visualization^(7)^. Other studies highlight the importance of overlaying thermographic and anatomical images for clearer ROI visualization. However, the equipment used in this study did not allow for this procedure, requiring a color scale that helped identify anatomical contours^(22)^.

The maximum, mean, and minimum temperatures determined the best interrater and intrarater agreement and compared MB with NB children. The method demonstrated satisfactory (moderate to excellent) interrater agreement for maximum temperatures, suggesting the use of maximum temperature to analyze the periorbital region. Specifically, the RU and UL ROIs had the best interrater agreement for maximum temperatures. These regions correspond to the upper right and left arcs, respectively, and may be influenced by the vascularization of the supraorbital artery, with the warmer regions located within these areas^(20)^. Minimum temperatures, on the other hand, had a poor agreement, and it is suggested that these should either not be used or be interpreted with caution in analyses. The literature typically uses maximum and mean temperatures^(22,23)^, consistent with the findings of this study. Intrarater agreement was satisfactory for most measures – except for the minimum and mean UL ROI temperatures, which were poorly correlated between one examiner's measurements.

A study analyzed the temperatures of the ocular surface, eyelid, and periorbital region of volunteers without ocular changes and diagnosed with Sjögren’s syndrome, evaporative dry eye, and dry eyes due to aqueous deficiency. Its method selected three ROIs with the ellipse tool in the orbital surface, eyelid, and periorbital region. Subjects without ocular changes had higher mean temperatures in all ROIs – the superior nasal region in the periorbital area had the highest temperature among the nine regions analyzed^(23)^. This finding is consistent with the results of the present study and reinforces the possible influence of the vascularization of the supraorbital artery on the temperature measured within this ROI.

When analyzing whether the breathing mode interferes with the periorbital surface temperature of the region, no difference was found between MB and NB individuals. This contradicts the research hypothesis for all temperatures analyzed, except for the standardized minimum UL ROI temperature, which was lower in the MB group. However, since this measure did not have good intrarater or interrater agreement, it is believed that this isolated finding is not enough to conclude that there is a difference between the study groups.

On the other hand, ocular edema was associated with the absolute minimum and mean UR ROI temperatures and absolute mean and maximum and standardized maximum LR ROI temperatures in MB children. The periorbital region has a complex blood supply system, and the literature indicates that MB people may have changes in the venous drainage of the eyelid region due to the edema in the nasal and paranasal mucosa, which results in blood stasis and the appearance of periorbital hyperchromia (dark circles)^(24)^. Furthermore, the periorbital region is spongy, and factors such as sleep disturbances, allergic processes, or salty diets can cause edema in this region^(24)^. The sample in the present study had four children with periorbital edema, all of whom had an allergy diagnosis, and their ROI temperatures were higher than in children without edema. The study by Ishimaru and Ishimaru^(25)^ showed that subjects with sinusitis had higher temperatures in the region due to the inflammatory process, as the accumulation of fluid raises the local temperature.

The presence of ocular itching was associated with the maximum UR ROI temperature. Ocular itching occurs due to processes that irritate the eye conjunctiva, primarily including allergic reactions affecting the ocular region or allergic processes in the nasal mucosa^(26,27)^. However, this isolated finding is limited to a single ROI, highlighting the need for further research with a larger sample size to evaluate its significance.

This study has limitations due to its exploratory nature and small sample size, but the findings indicate the relevance of continuing research with this population. It is important to address the cause of MB, as individuals with different diagnoses have specific clinical and physiological characteristics that may influence ROI temperatures. Additionally, sleep disorders in MB children require comprehensive evaluation using established methods such as polysomnography and supplementary tools like questionnaires. These approaches can elucidate aspects of sleep quality and its impact on the child’s life, which may affect their facial analysis.

The strengths of this study include the rigorous methodology regarding the inclusion of children in the groups, the homogeneity of the groups in terms of sex and age, and, particularly, the analysis of thermograms, which encompassed intrarater and interrater assessments. This is an innovative study, as no other research was found that evaluated the periorbital region to compare individuals with different breathing patterns. Studies like this must be conducted and refined, given that thermography is a painless, quick, and non-invasive technological tool that provides information about blood circulation on the body’s surface, which is related to various pathophysiological processes^(6)^.

## CONCLUSION

The proposed method demonstrated satisfactory intrarater and interrater agreement for maximum temperature. The respiratory mode was not associated with periorbital temperature. However, ocular edema was associated with analyzed temperatures in the MB group, with higher values.
