# Supplementary material for: Thermographic analysis of the periorbital region in mouth and nasal breathing children
Source: Codas. 2025 Feb 21;37(2):e20240151. doi: 10.1590/2317-1782/e20240151en (PMC11864766; doi:10.1590/2317-1782/e20240151en)
Supplement: Translation

# Translation: Análise termográfica da região periorbital em crianças respiradoras orais e nasais

**DOI:** 10.1590/2317-1782/e20240151pt

**Authors:** Júlia Ana Soares Silva, Amanda Freitas Valentim, Yasmim Carvalho Telson, Patrícia Vieira Salles, Mariana Souza Amaral, Ana Cristina Côrtes Gama, Letícia Paiva Franco, Andréa Rodrigues Motta, Renata Maria Moreira Moraes Furlan

**Affiliations:** 1 Faculdade de Medicina, Universidade Federal de Minas Gerais – UFMG - Belo Horizonte (MG), Brasil.; 2 Departamento de Fonoaudiologia, Faculdade de Medicina, Universidade Federal de Minas Gerais – UFMG - Belo Horizonte (MG), Brasil.; 3 Programa de Pós-graduação em Ciências Fonoaudiológicas, Universidade Federal de Minas Gerais – UFMG - Belo Horizonte (MG), Brasil.; 4 Departamento de Fonoaudiologia, Pontifícia Universidade Católica de Minas Gerais – PUC Minas - Belo Horizonte (MG), Brasil.; 5 Departamento de Otorrinolaringologia, Faculdade de Medicina, Universidade Federal de Minas Gerais – UFMG - Belo Horizonte (MG), Brasil.

**Keywords:** Termografia, Pálpebras, Respiração Bucal, Fonoaudiologia, Olho

## Abstract

**Objetivo:**

verificar se o modo respiratório interfere na temperatura superficial da região periorbital e propor um método para análise termográfica dessa região.

**Método:**

estudo exploratório observacional e transversal. As imagens termográficas de 14 crianças respiradoras orais e 14 respiradoras nasais foram coletadas e analisadas pelo programa Visionfy (Thermofy®, Brasil) com a escala de cor mama 1. Utilizou-se a ferramenta elipse para delimitação da região de interesse (ROI), região periorbital, e uma forma personalizada para subdividir a região de interesse em quatro arcos. Foram coletadas as temperaturas máxima, média e mínima (absolutas e normalizadas) dos arcos. Foram realizadas comparação inter e intra-avaliador e análise comparativa das temperaturas entre os grupos. No grupo de respiradores orais realizou-se a comparação entre as temperaturas e as variáveis presença de prurido, hiperemia, lacrimejamento e edema oculares.

**Resultados:**

a concordância intra-avaliador foi satisfatória para a maior parte das temperaturas analisadas. A concordância interavaliador, em geral, foi excelente para as temperaturas máximas, boa para as médias e ruim para as mínimas. Houve associação entre a presença de edema ocular e as temperaturas dos arcos superior e inferior direito, bem como entre a presença de prurido e a temperatura máxima do arco superior direito.

**Conclusão:**

o método proposto mostrou concordância intra e interavaliador satisfatória para as temperaturas máxima e média. Verificou-se associação entre edema ocular e as temperaturas analisadas no grupo de respiradores orais. O modo respiratório não apresentou associação com temperatura da região periorbital.

## INTRODUÇÃO

A respiração é uma função do sistema estomatognático que possui grande influência no desenvolvimento global do indivíduo, sendo que alterações nessa função podem afetar o desenvolvimento craniofacial, cognitivo e comportamental^(1)^. A respiração oral (RO) ocorre quando há substituição do padrão respiratório nasal pelo padrão oral ou misto por período igual ou superior a seis meses^(2,3)^. Diversos fatores podem ser precursores desse modo respiratório, os quais abrangem obstruções nasais, distúrbios alérgicos, alterações fisiológicas/anatômicas e fatores genéticos^(2,3)^. A principal etiologia é a rinite alérgica (RA), seguida de hipertrofia de adenoide, hipertrofia de amígdalas e desvio obstrutivo de septo nasal^(2)^.

Os principais sinais e sintomas incluem tensão diminuída dos músculos orofaciais, cansaço e irritabilidade, halitose, sonolência diurna e alterações nas funções da fala, mastigação e deglutição^(4)^. Dessa forma, a criança com RO necessita da avaliação multiprofissional para diagnóstico e tratamento adequados^(4)^. Além dos sinais e sintomas mais comumente encontrados, as alterações na qualidade do sono de crianças com RO também exigem atenção, em virtude da prevalência de alterações como ronco, dormir de boca aberta, apneia noturna, sialorreia e sono fragmentado^(5)^.

No campo da Fonoaudiologia, a avaliação da criança com RO demanda testes específicos que podem ser qualitativos ou quantitativos, contudo, há o predomínio de avaliações com técnicas qualitativas, como a observação do avaliador sobre as alterações miofuncionais^(1)^. Novas tecnologias têm sido sugeridas como potencialmente interessantes para auxiliar o fonoaudiólogo no processo de avaliação, dentre elas a termografia infravermelha, um recurso não invasivo, que permite ao profissional monitorar a atividade microcirculatória da superfície cutânea de forma rápida, segura e indolor, podendo, assim, identificar processos inflamatórios, alterações endócrinas e dos sistemas vascular, nervoso e musculoesquelético^(1,6,7)^.

A pele da região periorbital é a mais delgada do corpo humano e a sua anatomia conta com um sistema de irrigação vascular complexo que abrange a artéria carótida interna, a qual se ramifica em artéria oftálmica, e também a artéria carótida externa, ramificando-se em artérias infraorbital, facial e temporal superficial. As veias temporal superficial, angular e facial são as responsáveis pela drenagem venosa da pálpebra^(8)^. Estudos demonstram que a má qualidade do sono gera estresse oxidativo e correlaciona-se a alterações e envelhecimento da pele, portanto comumente é associado a esse fato a hipercromia periorbital, que é definida como uma estase dos vasos sanguíneos, causando a coloração diferenciada na região periorbital^(9,10)^. Tal sinal é também frequentemente observado em crianças com a RO^(10)^.

Dessa forma, o objetivo deste estudo é verificar se o modo respiratório interfere na temperatura superficial da região periorbital e propor um método para análise termográfica dessa região. A hipótese do estudo é que há diferença na temperatura da região periorbital entre indivíduos respiradores orais e nasais, com os respiradores nasais apresentando maior temperatura nessa região, em decorrência da melhor irrigação sanguínea^(9)^.

## MÉTODO

Trata-se de um estudo do tipo exploratório observacional e transversal, aprovado pelo Comitê de Ética em Pesquisa da Universidade Federal de Minas Gerais (UFMG), sob o parecer 3.695.491. Todos os pais ou responsáveis legais pelas crianças que participaram do estudo assinaram o Termo de Consentimento Livre e Esclarecido.

Participaram do estudo 28 crianças de quatro a 11 anos (média de idade de 7,62 anos, desvio-padrão de 2,19 anos), de ambos os sexos, divididas em dois grupos:

1- Grupo RO: crianças respiradoras orais, sendo 10 do sexo masculino e quatro do sexo feminino (n=14);
2- Grupo RN: crianças respiradoras nasais, sendo 10 do sexo masculino e quatro do sexo feminino (n=14).

As crianças respiradoras orais foram recrutadas no Ambulatório do Respirador Oral, do Hospital das Clínicas da UFMG. As crianças respiradoras nasais foram recrutadas no Ambulatório de Fonoaudiologia do Hospital das Clínicas da UFMG, dentre aquelas que realizavam atendimento nas áreas de Linguagem e Audiologia, bem como outras crianças foram convidadas por meio de contato direto realizado pelos pesquisadores.

Os critérios de inclusão para ambos os grupos foram: responder aos protocolos de anamnese e/ou exame clínico para identificação de crianças respiradoras orais^(2)^ e ter idade entre dois e 11 anos.

Constituíram os critérios de exclusão para ambos os grupos: uso de adornos ou bandagens sobre a face; ter tomado banho ou utilizado secador de cabelo duas horas antes do exame; ter aplicado produtos sobre a face no dia do exame; ter consumido cafeína quatro horas antes ao exame; ter se alimentado duas horas antes ao exame; apresentar alteração física, neurológica ou cognitiva que impedisse ou dificultasse a colaboração durante a realização do exame; ter utilizado o celular próximo à face duas horas antes ao exame; ter realizado exercícios físicos, acupuntura, massagens, estimulação elétrica ou exposição prolongada ao sol no dia do exame^(11)^; e apresentar ferimentos, inflamações ou processos cicatriciais na área de interesse da coleta. Também foram excluídos aqueles que utilizaram vasoconstritores ou corticoides nasais no dia da coleta. Esses critérios foram analisados por meio de um questionário aplicado aos pais.

O critério estabelecido para classificação como respirador oral (RO) foi o diagnóstico multidisciplinar da equipe do Ambulatório do Respirador Oral do Hospital das Clínicas da UFMG, composta por otorrinolaringologistas, alergologista, ortodontista e fonoaudiólogo. Os pacientes classificados como obstrutivos foram aqueles que apresentaram hipertrofia adenoideana, ou seja, adenoide maior que 70%^(12)^, obstruindo o espaço aéreo nasofaríngeo, com presença ou não de hipertrofia amigdaliana, além das crianças com a presença de cornetos inferiores hipertrofiados que ocupavam mais de 50% da fossa nasal^(2)^, visualizadas na fibronasolaringoscopia e rinoscopia. Os pacientes classificados como alérgicos foram aqueles que apresentaram teste cutâneo (Teste de puntura ou Prick Test) positivo^(13)^. Foram coletados dos prontuários dos participantes respiradores orais, dados referentes à causa do modo respiratório oral (presença de obstrução de via aérea e/ou de alergias), bem como dados referentes à região periorbital, relacionados à presença de hiperemia, lacrimejamento, edema e prurido ocular. Tais informações periorbitais são registradas nas avaliações realizadas pelos otorrinolaringologistas.

Para a classificação da criança como respiradora nasal, foi aplicada a prova de possibilidade de uso nasal^(14)^, sendo realizada a observação do posicionamento labial durante a postura habitual, em que a criança deveria manter o vedamento labial sem tensão. Foi aplicado, também, o protocolo de anamnese e exame clínico para identificação de crianças respiradoras orais^(2)^, cujas respostas aos parâmetros determinados identificam a criança como uma respiradora nasal caso apresente, no máximo, um sinal maior ou um sinal maior associado a um sinal menor. Ademais, foi utilizada a Escala de Distúrbios de Sono em Crianças^(15)^, cuja finalidade foi garantir que as crianças respiradoras nasais não possuíssem distúrbios do sono associados, sendo que a pontuação deveria ser menor ou igual a 39.

A coleta dos dados foi realizada no Observatório de Saúde Funcional em Fonoaudiologia da Faculdade de Medicina da UFMG, em ambiente controlado, cuja temperatura se encontrava entre 20ºC e 23ºC e a umidade relativa do ar entre 40% e 70%, sendo que os dados foram registrados pelo termohigrômetro da marca Testo®, modelo 622. Ademais, os pacientes permaneceram no ambiente por 15 a 20 minutos antes da avaliação termográfica, para estabilizar a temperatura. Para normalização dos valores de temperatura, foi realizada a medição da temperatura timpânica de cada participante por meio do termômetro da marca Prosnubl®. Para determinar as condições de padronização para o estudo, foi utilizado o guia prático da Academia Americana de Termologia como referência^(11)^.

Para a avaliação termográfica foi utilizada câmera FLIR A315®, cujas especificações estão descritas no Quadro 1^(16)^:

**Quadro 1:** Especificações da câmera FLIR A315

Modelo	Flir A315
Distância Focal da Lente	18 mm
Resolução	320 x 240 pixels
FOV	25° x 18,8°
IFOV	1,36 mrad
Faixa do Espectro	7,5 a 13 μm
Faixa de Medição de Temperatura	-20°C a 120°C / 0 a 350°C
Incerteza	±2°C ou ±2%

Legenda: FOV = Field of view; IFOV = instantaneous field of view

Para a avaliação termográfica, as crianças foram posicionadas sentadas em uma cadeira, pernas alinhadas ao solo, a 90° em relação ao tronco e cabeça em posição habitual. Foi solicitado que a criança utilizasse um prendedor de cabelo e/ou touca, retirasse adornos, como brincos e colares, e, obrigatoriamente, retirasse a máscara de proteção facial.

A câmera foi posicionada em um tripé, com 90° de angulação em relação ao chão, distância do encosto da cadeira de um metro e altura ajustável de acordo com a estatura da criança. As imagens térmicas foram repetidas três vezes com a criança em posição frontal com os lábios em posição habitual.

As medições coletadas foram armazenadas em uma pasta particular na memória do computador e os dados transpostos para uma planilha no Excel. A análise das imagens foi feita por meio do programa *Visionfy (Thermofy*®, *Brasil)*, considerando a emissividade da pele humana como 0,98^(11)^ e a escala de cor *mama 1* foi selecionada para permitir uma visualização clara da área de interesse, com uma janela de temperatura entre 25ºC e 37ºC. Nessa paleta de cor, o predomínio de cinza claro refere-se às temperaturas mais frias, o cinza escuro e o preto são temperaturas médias e o roxo e o amarelo são temperaturas mais elevadas.

A região de interesse, região periorbital, nos termogramas, foi selecionada por meio da ferramenta elipse, a qual permite obter a temperatura média dentro de uma região oval/elíptica, cujo perímetro passa por pontos de referência específicos, e pela forma personalizada, a qual delimita a região periorbital. Os seguintes limites foram respeitados para selecionar a região periorbital (Figura 1): limite superior (região do supercílio onde está a sobrancelha); limite inferior (inserção da pálpebra inferior abaixo da borda inferior orbital, junção da pálpebra ao tecido mais denso da bochecha, visualizado por uma região mais escura); limite lateral (região mais escura, fora das comissuras oculares, correspondendo à região de conexão entre a pálpebra inferior e superior)^(8,17)^.

**Figura 1:** Definição das áreas de interesse

Legenda: ROI = região de interesse; PoD = região periorbital direita; PoE = região periorbital esquerda; OD = região da órbita direita; OE = região da órbita esquerda; SD = arco superior da órbita direita; SE = arco superior da órbita esquerda; ID = arco inferior da órbita direita; IE = arco inferior da órbita esquerda

Ao obter a delimitação da área referente à região periorbital, inseriu-se uma linha horizontal que passou em cima das comissuras oculares e, assim, uma elipse foi inserida para demarcar a região de interesse 1, representada pelas regiões periorbitais direita (ROI PoD) e esquerda (ROI PoE), a qual foi utilizada como referência para inserir as demais formas, juntamente com a linha horizontal. Além disso, foi demarcada a região de interesse 2, representada pelas regiões da órbita direita (ROI OD) e esquerda (ROI OE), cuja demarcação permitiu excluir as temperaturas oculares.

A partir dessa seleção, as demais ROIs foram demarcadas com a forma personalizada, sendo: o arco superior da órbita direita e da órbita esquerda do participante, correspondente aos ROI SD e ROI SE, respectivamente; o arco inferior da órbita direita e da órbita esquerda do participante, correspondente aos ROI ID e ROI IE, respectivamente.

Após a seleção das formas, obtiveram-se os dados das temperaturas máxima, média e mínima absolutas e também normalizadas das ROIs SD, ID, SE e IE, separadamente. A obtenção das temperaturas normalizadas teve como objetivo padronizar os valores encontrados, de acordo com a relação entre o metabolismo humano e as condições ambientais às quais o corpo está exposto. Para tanto, utilizou-se a equação da temperatura normalizada^(18)^ (Quadro 2), a qual fornece o valor no intervalo de zero a um, permitindo uma análise homogênea e evitando a interferência da temperatura ambiente no resultado.

**Quadro 2:** Equação da temperatura normalizada

Temperatura normalizada =	Temperatura média do ponto de interesse – Temperatura ambiente
	Temperatura timpânica – Temperatura ambiente

A marcação da área de interesse e obtenção das temperaturas foram realizadas por dois avaliadores independentes, sendo que ambos replicaram 20% das análises em dois momentos diferentes.

Utilizaram-se a média e o desvio-padrão das temperaturas das regiões de interesse na análise descritiva. Os dados foram analisados utilizando o software Stata versão 13. A análise comparativa das idades e das temperaturas entre os grupos foi realizada pelo teste de Mann Whitney (teste não paramétrico) e o teste Kruskal-Wallis foi utilizado no grupo de respiradores orais para comparação entre as temperaturas e as variáveis presença de prurido, hiperemia, lacrimejamento e edema oculares. O nível de significância adotado em todas as análises foi de 5%. A comparação inter e intra-avaliador foi realizada por meio do coeficiente de correlação intraclasse. O ICC foi classificado como ruim (<0,5); moderado (0,5 a <0,75); bom (0,75 a<0,9) e excelente (≥0,9)^(19)^.

## RESULTADOS

A amostra não apresentou diferença estaticamente significante de idade entre os grupos RO e RN, como indicado na Tabela 1.

**Tabela 1:** Comparação de idade entre respiradores orais e nasais

**Grupo**	**Média**	**Desvio-padrão**	**Mediana**	**Valor de p**
RN	7,81	2,26	8,20	0,549
RO	7,43	2,19	7,95	
Total	7,62	2,19	8,00	0,549

Legenda: RN = respirador nasal; RO = respirador oral; p = probabilidade de significância (Teste de Mann Whitney)

O grupo de respiradores orais contou com sete crianças alérgicas, duas obstrutivas, quatro classificadas como mistas (obstrutivas e alérgicas) e uma criança classificada como habitual. Ademais, os dados da região periorbital indicaram que 11 crianças apresentaram prurido ocular, sete apresentaram hiperemia, seis apresentaram lacrimejamento e quatro apresentaram edema.

A concordância interavaliador é descrita na Tabela 2. Verificou-se que as temperaturas máximas apresentaram concordância moderada a excelente, as temperaturas médias obtiveram concordância ruim a boa e as mínimas classificadas entre ruim a moderada.

**Tabela 2:** Comparação interavaliador das medidas de temperaturas (ºC)

**Medidas de temperatura (ºC)**	**ICC**	**Grau da correlação**
Máxima da ROI SD	0,984	Excelente
Mínima da ROI SD	0,084	Ruim
Média da ROI SD	0,847	Bom
Máxima da ROI ID	0,730	Moderado
Mínima da ROI ID	0,208	Ruim
Média da ROI ID	0,474	Ruim
Máxima da ROI SE	0,998	Excelente
Mínima da ROI SE	0,475	Ruim
Média da ROI SE	0,859	Bom
Máxima da ROI IE	0,687	Moderado
Mínima da ROI IE	0,502	Moderado
Média da ROI IE	0,616	Moderado

Legenda: ICC = Coeficiente de Correlação Intraclasse; ROI = região de interesse; SD = arco superior da órbita direita; SE = arco superior da órbita esquerda; ID = arco inferior da órbita direita; IE = arco inferior da órbita esquerda.

Na concordância intra-avaliador (Tabela 3), o avaliador 1 apresentou concordância boa a excelente para as temperaturas máximas, enquanto, para as temperaturas médias e mínimas, a concordância foi ruim a excelente. O avaliador 2 obteve concordância moderada a excelente para as temperaturas máximas e médias, enquanto as mínimas tiveram concordância boa a moderada.

**Tabela 3:** Comparação intra-avaliadores das medidas de temperaturas

**Medidas de temperatura**	**Avaliador 1 (n=6)**		**Avaliador 2 (n=6)**	
	**ICC**	**Grau da correlação**	**ICC**	**Grau da correlação**
Máxima da ROI SD	0,998	Excelente	0,998	Excelente
Mínima da ROI SD	0,977	Excelente	0,706	Moderado
Média da ROI SD	0,976	Excelente	0,958	Excelente
Máxima da ROI ID	0,905	Excelente	0,744	Moderado
Mínima da ROI ID	0,870	Bom	0,814	Bom
Média da ROI ID	0,935	Excelente	0,629	Moderado
Máxima da ROI SE	0,998	Excelente	0,992	Excelente
Mínima da ROI SE	0,101	Ruim	0,513	Moderado
Média da ROI SE	0,351	Ruim	0,959	Excelente
Máxima da ROI IE	0,896	Bom	0,714	Moderado
Mínima da ROI IE	0,966	Excelente	0,544	Moderado
Média da ROI IE	0,953	Excelente	0,514	Moderado

Legenda: ICC = Coeficiente de Correlação Intraclasse; ROI = região de interesse; SD = arco superior da órbita direita; SE = arco superior da órbita esquerda; ID = arco inferior da órbita direita; IE = arco inferior da órbita esquerda

Foi realizada também a comparação das temperaturas entre os grupos de RO e RN (Tabela 4), observou-se que houve diferença significativa somente para a temperatura mínima normalizada da ROI SE. Observou-se, ainda, que a maior diferença de temperatura entre grupos ocorreu para as temperaturas mínimas da ROI SE, em que a diferença entre as médias dos grupos RO e RN foi de 0,69ºC, e da ROI IE com diferença de 0,62ºC.

**Tabela 4:** Distribuição das medidas de temperaturas (°C) dos grupos de respiradores orais e nasais

**Medidas de temperatura**	**Grupo RO**		**Grupo RN**		**Valor de p**
	**Média**	**Desvio-padrão**	**Média**	**Desvio-padrão**	
Máxima da ROI SD	35,22	0,63	35,50	0,55	0,265
Mínima da ROI SD	32,50	0,49	32,71	0,68	0,461
Média da ROI SD	33,81	0,51	34,12	0,55	0,167
Máxima normalizada da ROI SD	0,93	0,04	0,94	0,03	0,370
Mínima normalizada da ROI SD	0,75	0,03	0,76	0,04	0,395
Média normalizada da ROI SD	0,84	0,03	0,85	0,02	0,160
Máxima da ROI ID	34,64	0,85	34,94	0,70	0,270
Mínima da ROI ID	31,95	1,03	32,21	0,84	0,490
Média da ROI ID	33,43	0,85	33,64	0,55	0,635
Máxima normalizada da ROI ID	0,89	0,05	0,90	0,04	0,358
Mínima normalizada da ROI ID	0,71	0,06	0,72	0,04	0,709
Média normalizada da ROI ID	0,81	0,04	0,82	0,02	0,865
Máxima da ROI SE	35,37	0,47	35,47	0,42	0,804
Mínima da ROI SE	32,04	0,79	32,73	0,70	0,062
Média da ROI SE	33,73	0,58	34,12	0,50	0,085
Máxima normalizada da ROI SE	0,94	0,03	0,94	0,03	0,991
Mínima normalizada da ROI SE	0,72	0,04	0,76	0,04	0,038*
Média normalizada da ROI SE	0,83	0,03	0,85	0,02	0,101
Máxima da ROI IE	34,99	0,78	35,03	0,59	0,972
Mínima da ROI IE	31,49	0,93	32,11	0,87	0,125
Média da ROI IE	33,44	0,91	33,62	0,53	0,769
Máxima normalizada da ROI IE	0,91	0,04	0,91	0,04	0,692
Mínima normalizada da ROI IE	0,68	0,05	0,72	0,05	0,087
Média normalizada da ROI IE	0,81	0,05	0,82	0,03	0,865

* p≤0,05

Legenda: RN = respirador nasal; RO = respirador oral; ROI = região de interesse; SD = arco superior da órbita direita; SE = arco superior da órbita esquerda; ID = arco inferior da órbita direita; IE = arco inferior da órbita esquerda; p = probabilidade de significância (Teste de Mann Whitney)

Na Tabela 5 é apresentada a associação entre as temperaturas absolutas e normalizadas das seguintes variáveis do grupo de respiradores orais: presenças de prurido, hiperemia, lacrimejamento e edema oculares. Foi verificado que houve associação entre a presença de edema ocular e as temperaturas mínima e média absolutas da ROI SD e temperaturas máxima e média absolutas e máxima normalizada da ROI ID. Ademais, a variável prurido ocular apresentou associação com a temperatura máxima normalizada da ROI SD.

**Tabela 5:** Associação entre as medidas de temperatura e a presença de edema ocular, prurido ocular, hiperemia ocular e lacrimejamento

Medidas de temperatura	Edema ocular			Prurido ocular			Hiperemia ocular			Lacrimejamento		
	Média	DP	Valor de p	Média	DP	Valor de p	Média	DP	Valor de p	Média	DP	Valor de p
Máxima da ROI SD	35,67	0,18	0,066	35,35	0,59	0,139	35,37	0,68	0,277	35,29	0,71	0,796
Mínima da ROI SD	32,89	0,16	0,048*	32,5	0,52	0,938	32,55	0,51	0,655	32,51	0,55	0,897
Média da ROI SD	34,22	0,19	0,048*	33,89	0,51	0,312	33,89	0,54	0,565	33,83	0,57	0,796
Máxima normalizada da ROI SD	0,95	0,01	0,203	0,94	0,03	0,043*	0,93	0,03	0,225	0,94	0,05	0,519
Mínima normalizada da ROI SD	0,76	0,01	0,09	0,75	0,03	0,938	0,75	0,02	0,949	0,75	0,03	0,796
Média normalizada da ROI SD	0,85	0,01	0,066	0,84	0,03	0,052	0,84	0,03	0,406	0,84	0,04	0,796
Máxima da ROI ID	35,33	0,45	0,034*	34,75	0,88	0,243	34,87	1,04	0,142	34,61	0,96	0,897
Mínima da ROI ID	32,45	0,84	0,258	31,97	1,08	0,586	32,13	1,03	0,338	31,95	1,03	0,897
Média da ROI ID	34,11	0,32	0,034*	33,52	0,88	0,312	33,67	0,99	0,142	33,45	0,97	0,897
Máxima normalizada da ROI ID	0,92	0,03	0,034*	0,9	0,05	0,073	0,9	0,06	0,142	0,89	0,06	0,699
Mínima normalizada da ROI ID	0,73	0,06	0,396	0,71	0,06	0,697	0,72	0,06	0,565	0,71	0,06	0,897
Média normalizada da ROI ID	0,84	0,02	0,066	0,82	0,04	0,139	0,82	0,05	0,18	0,81	0,05	0,699
Máxima da ROI SE	35,66	0,23	0,12	35,42	0,46	0,243	35,54	0,42	0,142	35,47	0,42	0,699
Mínima da ROI SE	32,58	0,46	0,09	31,99	0,72	0,484	32,31	0,61	0,225	32	0,59	0,606
Média da ROI SE	34,11	0,24	0,12	33,73	0,51	0,586	33,84	0,55	0,482	33,69	0,5	0,366
Máxima normalizada da ROI SE	0,94	0,02	0,322	0,94	0,03	0,073	0,94	0,02	0,18	0,95	0,03	0,245
Mínima normalizada da ROI SE	0,74	0,02	0,12	0,72	0,04	0,586	0,73	0,03	0,18	0,72	0,04	0,796
Média normalizada da ROI SE	0,84	0,01	0,203	0,83	0,02	0,697	0,83	0,03	0,565	0,83	0,03	1
Máxima da ROI IE	35,53	0,41	0,089	35	0,85	0,815	35,17	0,99	0,179	34,88	0,94	0,518
Mínima da ROI IE	31,81	0,33	0,671	31,38	0,9	0,312	31,52	0,88	0,949	31,34	1,05	0,699
Média da ROI IE	33,93	0,47	0,203	33,45	0,95	0,815	33,55	1,13	0,406	33,35	1,13	0,519
Máxima normalizada da ROI IE	0,94	0,03	0,157	0,92	0,05	0,312	0,92	0,05	0,18	0,91	0,05	0,796
Mínima normalizada da ROI IE	0,69	0,02	1	0,68	0,06	0,312	0,68	0,04	0,565	0,67	0,07	0,519
Média normalizada da ROI IE	0,83	0,03	0,396	0,81	0,05	0,484	0,81	0,06	0,406	0,81	0,06	0,796

* p≤0,05

Legenda: p = probabilidade de significância (Teste Kruskal-Wallis); ROI = região de interesse; SD = arco superior da órbita direita; SE = arco superior da órbita esquerda; ID = arco inferior da órbita direita; IE = arco inferior da órbita esquerda; DP = desvio-padrão

## DISCUSSÃO

Por meio desta pesquisa foi possível desenvolver um método para registro e análise termográfica da região periorbital e obtenção das temperaturas superficiais de crianças respiradoras orais e nasais. O uso da termografia na região orbital e periorbital tem se consolidado como material de estudo de diversos pesquisadores na área de Oftalmologia para diagnóstico de processos inflamatórios, doenças oculares, processos alérgicos e identificação de padrões de temperatura ocular^(20)^. A vantagem do método consiste na utilização de um instrumento de medição indireta da temperatura que, por não ter contato com o paciente, não causa desconforto e não interfere na temperatura desse^(7)^.

O método utilizado neste trabalho, para determinação das regiões de interesse, permitiu às pesquisadoras a avaliação somente da região periorbital, pois tomou-se o cuidado de retirar a participação de estruturas adjacentes que poderiam influenciar nas temperaturas analisadas, já que a região da órbita e cílios apresentam temperaturas mais frias^(21)^. Estudos evidenciam que a região central da superfície orbital é a mais fria, visto que essa área possui baixa vascularização e sua temperatura é influenciada pela lubrificação natural do olho e pelo ambiente^(20)^.

As regiões de interesse abordadas no presente estudo basearam-se na anatomia facial dos indivíduos. A utilização da escala de *cor mama 1* permitiu melhor visualização das regiões de interesse, por se aproximar da visualização de imagens anatômicas^(7)^. Outros estudos abordam a importância da utilização da sobreposição de imagens termográficas e anatômicas para melhor visualização das regiões de interesse, entretanto o equipamento utilizado não possibilitou que as pesquisadoras usassem esse procedimento, por isso foi importante a escolha de uma escala de cor que permitisse a identificação de contornos da anatomia dos indivíduos^(22)^.

As temperaturas coletadas foram a máxima, média e mínima para verificação das que possuíssem melhor concordância inter e intra-avaliadores e que permitissem a comparação e diferenciação entre respiradores orais e nasais. O método apresentou concordância interavaliador satisfatória (moderada a excelente) para a temperatura máxima, portanto sugere-se o uso da temperatura máxima nas análises da região periorbital. As ROIs SD e SE, em especial, foram as regiões com as melhores concordâncias para a temperatura máxima, entre os examinadores. Estas regiões correspondem aos arcos superiores direito e esquerdo, respectivamente. Uma possível explicação para este achado pode estar atrelada ao fato de que tais ROIs incluem a região do canto nasal de cada hemiface, que sofre influência da vascularização da artéria supraorbital, portanto a região mais aquecida encontra-se dentro destas regiões de interesse^(20)^. Já as temperaturas mínimas foram as menos concordantes, portanto sugere-se que não sejam utilizadas ou que sejam interpretadas com cautela nas análises. A literatura utiliza como padrão as temperaturas máxima e média^(22,23)^, o que vai ao encontro dos achados da presente pesquisa. A concordância intra-avaliador foi satisfatória para a maioria das medidas. Apenas as temperaturas mínima e média da ROI SE apresentou correlação ruim entre as medidas realizadas por um dos examinadores.

Um estudo buscou analisar as temperaturas da superfície ocular, pálpebra e região periorbital de voluntários sem alterações oculares e com diagnóstico de síndrome de Sjögren, olho seco evaporativo e olhos secos por deficiência aquosa. O método utilizado baseou-se na escolha de três regiões de interesse, selecionadas pela ferramenta elipse, na área da superfície orbital, pálpebra e área periorbital. Em todas as regiões de interesse, os sujeitos sem alteração ocular obtiveram maiores valores de temperatura média, sendo a região de interesse denominada nasal superior, localizada na área periorbital, a que apresentou a temperatura mais elevada entre as nove regiões analisadas^(23)^, o que concorda com os achados da presente pesquisa e reforça a possível influência da vascularização da artéria supraorbital na temperatura medida dentro dessa região de interesse.

Ao analisar se o modo respiratório interfere na temperatura superficial da região periorbital, verificou-se ausência de diferença entre os indivíduos respiradores orais e nasais, o que contraria a hipótese da pesquisa para todas as temperaturas analisadas, com exceção da temperatura mínima normalizada da ROI SE. Nesse caso a temperatura foi menor para o grupo de respiradores orais, porém, considerando que esta medida não teve boa concordância intra e interavaliador, acredita-se que este achado isolado não seja suficiente para considerar que haja diferença entre os grupos estudados.

Por outro lado, dentre os respiradores orais, foi verificada associação entre presença de edema ocular e as temperaturas mínima e média absolutas da ROI SD e temperaturas média e máxima absolutas e máxima normalizada da ROI ID. A região periorbital possui um sistema de irrigação sanguínea complexo, na literatura verifica-se que respiradores orais podem apresentar alteração na drenagem venosa da região palpebral, devido à presença de edema na mucosa nasal e paranasal, o que resulta na estase sanguínea e aparecimento da hipercromia periorbital (olheiras)^(24)^. Além disso, a região periorbital apresenta uma característica esponjosa, sendo que fatores como alterações do sono, processos alérgicos ou alimentação com excesso de sal podem provocar edemas nessa região ^(24)^. A amostra do presente estudo foi composta por quatro crianças com edema periorbital e todas apresentaram o diagnóstico de alergia, outrossim as temperaturas das regiões de interesse foram mais elevadas do que em crianças sem a presença de edema. O estudo de Ishimaru e Ishimaru^(25)^ evidenciou que sujeitos com presença de sinusite apresentavam temperatura mais elevada na região devido ao processo inflamatório, visto que o acúmulo de líquido aumenta a temperatura local.

A presença de prurido ocular apresentou associação com a temperatura máxima da ROI SD. O prurido ocular ocorre devido à presença de processos que geram irritação na conjuntiva do olho, os quais abrangem, principalmente, alterações alérgicas que afetam a região ocular ou processos alérgicos da mucosa nasal^(26,27)^. Este achado isolado, entretanto, é restrito a uma única região de interesse, sendo necessárias novas pesquisas, com maior número de indivíduos, que avaliem a sua pertinência.

O estudo possui limitações devido ao seu perfil exploratório e amostra reduzida, sendo que os resultados encontrados indicam a pertinência da continuidade das pesquisas com essa população. A causa da RO é uma variável importante a ser considerada, visto que indivíduos com diferentes diagnósticos apresentam características clínicas e fisiológicas específicas que podem influenciar na temperatura das regiões pesquisadas. Verifica-se, também, que os distúrbios do sono em crianças com RO demandam uma avaliação vasta, utilizando métodos consolidados, como a polissonografia e recursos adjacentes como questionários que irão elucidar aspectos da qualidade do sono e impacto na vida da criança e que podem afetar na análise facial desses indivíduos.

Como pontos fortes da pesquisa pode-se citar a metodologia criteriosa quanto à inclusão das crianças nos grupos, à homogeneidade dos grupos no que diz respeito ao sexo e idade e, especialmente, quanto à análise dos termogramas, incluindo as avaliações intra e interavaliadores. Trata-se de um estudo inovador, visto que não foram encontrados outros que avaliassem a região periorbital para comparação entre indivíduos com diferentes modos respiratórios. Estudos como este precisam ser conduzidos e aprimorados, visto que a termografia é um recurso tecnológico indolor, de rápida execução e não invasivo, que traz informações sobre a circulação sanguínea da superfície corporal, a qual guarda relação com diversos processos fisiopatológicos^(6)^.

## CONCLUSÃO

O método proposto mostrou concordância intra e interavaliador satisfatória para a temperatura máxima. O modo respiratório não apresentou associação com a temperatura da região periorbital. Outrossim, verificou-se associação entre edema ocular e temperaturas analisadas no grupo de respiradores orais, sendo estas com valores mais elevados.
